# Bisphenol A and Phthalates in Diet: An Emerging Link with Pregnancy Complications

**DOI:** 10.3390/nu12020525

**Published:** 2020-02-19

**Authors:** Tiziana Filardi, Francesca Panimolle, Andrea Lenzi, Susanna Morano

**Affiliations:** Department of Experimental Medicine, “Sapienza” University, Viale del Policlinico 155, 00161 Rome, Italy; tiziana.filardi@uniroma1.it (T.F.); francesca.panimolle@uniroma1.it (F.P.); andrea.lenzi@uniroma1.it (A.L.)

**Keywords:** gestational diabetes, diet, endocrine disruptors, endocrine-disrupting chemicals, pregnancy, bisphenol A, BPA, phthalates, pregnancy outcomes

## Abstract

Endocrine-disrupting chemicals (EDCs) are exogenous substances that are able to interfere with hormone action, likely contributing to the development of several endocrine and metabolic diseases. Among them, Bisphenol A (BPA) and phthalates contaminate food and water and have been largely studied as obesogenic agents. They might contribute to weight gain, insulin resistance and pancreatic β-cell dysfunction in pregnancy, potentially playing a role in the development of pregnancy complications, such as gestational diabetes mellitus (GDM), and adverse outcomes. Pregnancy and childhood are sensitive windows of susceptibility, and, although with not univocal results, preclinical and clinical studies have suggested that exposure to BPA and phthalates at these stages of life might have an impact on the development of metabolic diseases even many years later. The molecular mechanisms underlying this association are largely unknown, but adipocyte and pancreatic β-cell dysfunction are suspected to be involved. Remarkably, transgenerational damage has been observed, which might be explained by epigenetic changes. Further research is needed to address knowledge gaps and to provide preventive measure to limit health risks connected with exposure to EDCs.

## 1. Introduction

The prevalence of obesity and metabolic diseases has been increasing over the last decades, and a complex interaction between multiple environmental and genetic factors might explain this trend [1]. Remarkably, there is mounting evidence that several metabolic adult-onset conditions, such as metabolic syndrome, type 2 diabetes (T2D) and cardiovascular diseases (CVD), might take roots in a hostile intrauterine environment related to an unfavourable maternal diet or lifestyle [2]. 

Diabetes occurring in the second or third trimester of pregnancy is known as gestational diabetes mellitus (GDM) [3]. Over the last decades, the prevalence of this condition has dramatically grown, along with the epidemic spread of obesity [4]. Overall, the prevalence of GDM is largely influenced by ethnicity, ranging between 12.9% and 5.8%, and by the diagnostic criteria applied [5]. High body mass index (BMI) has a considerable impact on the risk of developing GDM, as well as first degree family history of T2D and advanced maternal age [6,7]. GDM contributes to adverse gestational outcomes, such as the increased rates of preterm delivery and caesarean section [8]. The most common neonatal complications include dystocia, neonatal hypoglycaemia, jaundice and acute respiratory distress syndrome [8,9]. Besides the recognized short-term consequences, there is rising concern about the negative consequences observed several years later, such as the high risk of T2D occurrence [10,11]. It is estimated that, in European countries, the cumulative incidence of T2D in mothers previously affected by GDM ranges from 2.1% to 35.7% in a follow-up time of 5.5 months–15 years [5]. There is consistent evidence in longitudinal studies that previous GDM is linked with an increased risk of obesity, metabolic syndrome and T2D, not only in mothers but also in offspring [12,13,14]. Furthermore, children of mothers previously affected by GDM exhibit increased rates of CVD in adulthood, even of early onset [15,16]. 

Interestingly, a suboptimal milieu in utero, especially in the context of a particular window of susceptibility, might contribute to the development of pathological conditions, even with a long latency. For instance, in pregnancy complicated by GDM, foetal hyperglycaemia and hyperinsulinemia might influence vascular gene expression, resulting in endothelial dysfunction and contributing to the increased CVD risk in offspring [17]. In addition to the possible role played by endogenous factors, in recent years, increasing attention has been focused on the suspected contribution of environmental chemicals exposure in the development of metabolic and endocrine conditions, specifically during pregnancy [2]. According to the World Health Organization (WHO) definition, an endocrine disruptor (ED) is “an exogenous substance or mixture that alters function(s) of the endocrine system and consequently causes adverse health effects in an intact organism, or its progeny, or (sub) populations” [18]. Humans and animals come constantly into contact with endocrine-disrupting chemicals (EDCs), which are thought to interfere with hormone action at different stages. To date, plenty of substances are suspected to have a negative impact on human health, although only a few have been extensively evaluated in proper studies. Over the last decades, an association between EDCs and the development of metabolic diseases has emerged, and there is rising concern about the risk of adverse health outcomes, which might be largely underestimated. 

## 2. Endocrine Disruptors in Diet

EDCs are ubiquitous and extensively pollute food and water. Industrial processes of food production allow them to unintentionally enter the food chain and to accumulate in wildlife and in humans. Almost 800 chemicals are suspected to interfere with endocrine functions and, among them, bisphenol A (BPA) and phthalates have been broadly studied as “obesogenic” factors [18]. 

BPA is a chemical compound obtained from a reaction of condensation between phenol and acetone. It is considered the first synthetic estrogen, although without a steroid structure, as it does not include the phenanthrene nucleus [19]. Due to its property of acting as a linker between chemicals, BPA is adopted by the chemical industry to produce plastic polymers.

Phthalates are diesters of phthalic acid classified into high and low molecular weight phthalates. The first category includes several compounds that are largely adopted to make plastic more flexible and durable. Among them, the most commonly employed additive is di(2-ethylhexyl) phthalate (DEHP). Low molecular weight phthalates are mainly used in personal care products and cosmetics, but they are also widespread in insecticides and in food packaging plastic [20]. In particular, diethyl phthalate (DEP) is one of the major phthalates in commerce [20]. BPA and phthalates are therefore widely diffused, due to the considerable volume of plastic production [21]. Basically, plastic materials for food storage, such as bottles and containers, are the main source of these chemicals. Although released even at room temperature, cooling and heating considerably facilitate the leaching of chemicals from containers, resulting in the contamination of food and beverages [22,23]. Another major source of BPA is the inner layer of cans (made of epoxy resins). As a result, products stored in cans and plastic casings have the highest concentrations of BPA independently of the specific nutrient category. Fish, vegetables and dairy products which are not packed in plastic containers or cans have therefore low concentrations of BPA and phthalates [24,25,26]. Conversely, ready-to-eat food stored in plastic bags is a major exposure source [27]. After ingestion, BPA is partially metabolized by the intestinal microbiota and largely absorbed in the intestinal tract [28]. BPA is transformed in the liver mainly by glucuronidation and, to a lesser extent, by sulfation, being eventually eliminated by the kidney [29]. Thus, BPA-glucuronide is the main metabolite of BPA in humans. As for phthalates, exposure to DEHP is reflected by the presence of its metabolites in urine, such as mono(2-ethylhexyl) phthalate (MEHP), mono(2-ethyl-5-hydroxyhexyl) phthalate, mono(2-ethyl-5-carboxypentyl) phthalate and mono(2-ethyl-5-oxohexyl) phthalate. Whereas the main urinary metabolite of DEP is mono-ethyl phthalate (MEP). Remarkably, the bioactivity of phthalate metabolites is superior to that of the original substance [29]. 

Since BPA does not accumulate in fat, a reduction in the intake of food stored in plastic materials and the adoption of BPA-free plastic containers considerably limit exposure [30,31]. In contrast, high molecular weight phthalates are more lipophilic and accumulate in fat food. Indeed, high concentrations of DEHP and BPA in dairy, meat and fast food products, such as hamburgers, have been reported [32,33]. Valvi et al. observed an inverse association between consumption of organic food and phthalate urinary levels in pregnant women [34]. Although evidence from clinical studies is still limited, a short course diet excluding food in plastic or cans was able to produce a significant drop in the urinary levels of BPA and DEHP [35].

BPA and phthalate concentrations have been evaluated in different biologic fluids at various stages of life. In the general population, urinary levels of BPA are reported to be on average 1.63 ng/mL in men and 1.12 ng/mL in women, whereas in serum BPA concentrations range between 0.3–4.4 ng/mL [36]. As regards phthalates, levels in urine vary among different countries, ranging from 1 to 100 μg/L [20]. Differences between ethnic groups might be explained by genetic polymorphisms in enzymes involved in biotransformation processes [37]. Overall, phthalate metabolites, mainly DEHP and DEP, have considerably lower concentrations in serum than in urine [38].

Pregnancy and childhood are particularly sensitive windows of susceptibility to chemicals, and exposure is potentially more harmful. Given the reduced ability to metabolize and eliminate BPA compared to adults, foetuses and children have considerably high levels of BPA in blood and urine [39,40]. A widespread exposure to EDCs has been consistently reported in pregnancy. Phthalate metabolites have been found in urine of about 98–100% of pregnant women [41,42]. In a sample of 378 pregnant women, at least 93% exhibited detectable concentrations of eight phthalate metabolites in urine between 18 and 22 weeks [43]. A positive association between phthalate concentrations and BMI in pregnancy has been reported in several studies [34,44,45,46,47,48], and higher phthalate levels in urine were observed in African American pregnant women compared to Caucasian [43,49,50]. In pregnant women, MEP is the predominant phthalate metabolite in urine, reaching a median concentration of 30 μg/L in most studies. Interestingly, maternal education and income were inversely related with phthalates levels, suggesting that sociocultural and lifestyle patterns might significantly influence exposure [34,43]. Data on urinary concentrations of phthalates in newborns are controversial. While some authors reported similar levels to those in the mothers [51], others observed two- or three times lower concentrations in offspring [52]. As for children, higher levels of urinary phthalates compared to adults have been found [53]. 

Many EDCs, such as high molecular weight phthalates and, to a lesser extent, low molecular weight phthalates, are quite lipophilic and are stored in adipose tissue [54]. Remarkably, almost all EDCs are able to cross the placenta, reaching the cord blood and the amniotic fluid [55,56]. They are also transferred from mother to child with lactation [57]. BPA in foetal circulation and amniotic fluid is almost 1–3 ng/mL, although in the latter, changes have been observed throughout pregnancy, averaging 8.3 ng/mL in the second trimester and then dropping to almost 1.1 ng/mL at the end of gestation [40]. In breastmilk, an average BPA concentration of 0.61 ng/mL has been reported [58]. As regards phthalates, amniotic fluid and breastmilk showed similar concentrations, generally far lower than those observed in urine and in serum [59].

## 3. General Aspects of EDCs

Endocrine functions are disrupted by EDCs through several complex mechanisms which are still not completely understood. Nuclear receptors (NR) are targeted by most of these substances, as well as steroid synthesis and metabolism [60,61]. NRs are located in the cytoplasm or nucleus in a monomeric state. The interaction with endogenous or exogenous ligands induces the translocation in the nucleus, the dimerization and the activation of gene transcription [62]. 

Several EDCs, including BPA and phthalate metabolites, are able to bind to estrogen receptors α (ERα) and β (ERβ). These receptors regulate the growth and the differentiation of many tissues, such as the female reproductive tract and the mammary gland [63]. 

Androgens intervene in the differentiation of male foetuses. EDCs, mainly pesticides but also bisphenols, have displayed anti-androgenic activities, interfering with the function of the androgen receptor (AR) [64]. 

Notably, the “obesogenic” effect attributed to EDCs has been linked to the activity of peroxisome proliferator-activated receptors (PPARs), which are crucially involved in lipid and glucose metabolism and energy homeostasis. Specifically, PPARγ are highly expressed in adipose tissue, and MEHP-induced activation of these receptors stimulates adipogenesis in vitro and in vivo [65,66]. The PPARα is principally expressed in the liver and in brown adipose tissue, and it is a target of MEHP as well [67].

EDCs disrupt thyroid hormone signalling, mainly acting as antagonists of thyroid receptors α (TRα) and β (TRβ), resulting in hypothyroidism and alterations of brain development [68].

The biotransformation of exogenous substances in the liver is regulated by the pregnane X receptor (PXR) and the constitutive androstane receptor (CAR). PXR induces the transcription of genes coding for cytochrome P450 and other enzymes involved in the clearance of xenobiotic substances. Notably, CAR regulates also lipid metabolism by eliminating cholesterol in the small intestine [69]. The activation of PXR and CAR pathways therefore protects the endocrine systems against EDCs. However, several substances, including bisphenols, are able to disrupt their activity, causing adverse effects [64]. Similarly, the aryl hydrocarbon receptor (AhR) exerts protective functions by sensing the presence of xenobiotic compounds and leading to the activation of cytochrome P450 enzymes. Interestingly, AhR can influence adipogenesis by altering PPARγ expression, and some “obesogenic” EDCs act specifically by disrupting this pathway [70].

Retinoid X receptors (RXR) are targeted by EDCs as well. These receptors are able to heterodimerize with other partners, such as PPARs, PXR, CAR, RARs and TRs. Thus, a multitude of adverse responses can be triggered by the exogenous ligand of RXRs, affecting human health [71].

Generally, the interaction between hormones and their receptors is not linear but sigmoidal [72,73]. When receptors are downregulated by high concentration of ligands, a U-shaped dose-response curve, implying high responses at low and high concentrations, or an inverted U-shaped curve, showing a stronger effect at medium doses, are frequently observed as well [72]. Despite their agonist and antagonist actions, EDCs are potentially able to modulate almost every aspect of hormone metabolism. Notably, the effects of EDCs exerted at low doses can be quite different from those induced by high doses, and it is fairly difficult to define a clear cut-off above which damage occurs. As EDCs can act at extremely low concentrations, their negative effects in real settings is often caused by a chronic low-dose exposure [74,75]. The U.S. Environmental Protection Agency (EPA) established for BPA a reference dose, or safe dose (i.e., the highest acceptable oral dose of a toxic substance), of 50 μg/kg/day, based on a “lowest observed adverse effect level” (LOAEL) of 50 mg/kg/day, whereas a “no observed adverse effect level” (NOAEL) has not emerged from toxicological studies [76]. As regards DEHP, the reference dose is 20 μg/kg/day, based on a LOAEL of 29 mg/kg/day, whereas the NOAEL is 5.8 mg/kg/day [20]. The estimated range exposure in humans is 0.4–5.0 µg/kg/day for BPA and 0.5–25.0 µg/kg/day for DEHP [76].

Another critical aspect of EDCs’ actions is that their impact on health might not be immediately evident. In light of this, early contact with a substance might lead to long-term damage. Accordingly, most adult diseases that are suspected of being related to EDCs might be a result of intrauterine life exposure. EDCs are able to modulate gene expression and directly modify the epigenome by DNA methylation [77,78]. Notably, epigenetic modifications can be transmitted throughout generations, and the transgenerational effects might become manifest only several years later [79,80]. 

Although it is broadly accepted that chemicals interfering with hormone pathways are therefore able to cause adverse outcomes, in the evaluation of the risks connected to exposure, other factors should be considered, such as dose and duration of exposure [73]. Additionally, the specific period of the life cycle in which exposure occurs is crucial for the prediction of adverse outcomes. An extremely sensitive window is the time between conception and birth, when critical cellular processes (such as replication and differentiation) and organ development take place [81]. 

Interestingly, gender differences have consistently emerged in several studies, suggesting that similar conditions of exposure to an EDC may lead to different clinical manifestations which might be explained by several factors, such as the different expression of receptors or enzymes in EDCs target tissues and organs between male and female sexes [82]. 

## 4. Effects of Gestational Exposure to BPA and Phthalates

**Figure 1:**
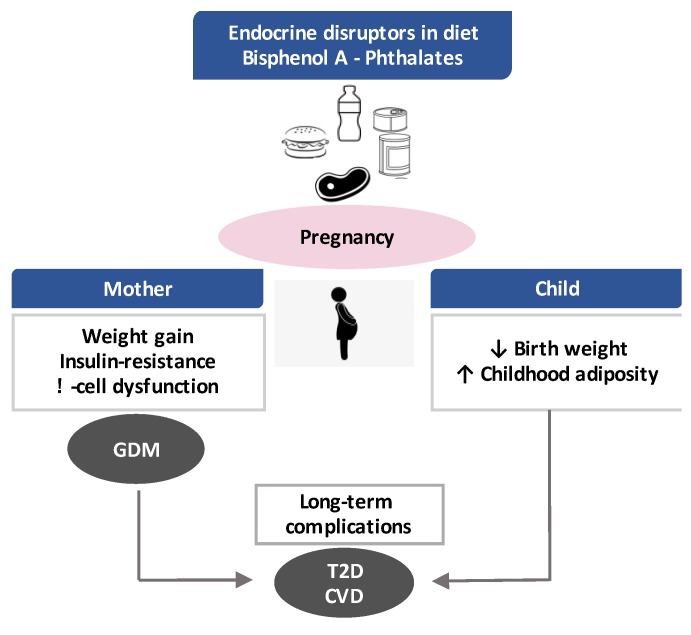
Endocrine disruptors in diet and pregnancy outcomes. GDM: gestational diabetes mellitus, T2D: type 2 diabetes and CVD: cardiovascular disease.

Exposure to BPA and phthalates during sensitive windows such as pregnancy can lead to metabolic dysfunction in the mother and interfere with foetal development. The disrupting effect might result in long-term consequences, both in the mother and in the offspring (Figure 1). 

### 4.1. Impact on the Mother and Risk of GDM

It is well-known that both adipose tissue and gestational tissues (such as the placenta) release a wide number of molecules that promote insulin resistance [83]. As a result, a progressive fall in insulin sensitivity is observed until the second trimester, even in healthy pregnancies. Nevertheless, the increase in insulin secretion by pancreatic β-cell prevents the development of GDM. GDM therefore occurs when the compensatory effect of the pancreatic β-cell is insufficient [84]. Furthermore, a low-grade pro-inflammatory state [85,86,87] has been described even in physiological pregnancy, and it was found to be enhanced in GDM, possibly contributing to the development of insulin resistance and adverse pregnancy outcomes [88,89,90]. 

BPA and phthalates seem to target several pathophysiological features of GDM, potentially playing a role in the pathogenesis of GDM. Indeed, they have been linked to weight gain, insulin resistance and pancreatic β-cell dysfunction. 

Gestational weight gain is a well-known risk factor for GDM [5]. In the Lifecodes cohort study, which enrolled 350 pregnant women, mean levels of maternal urinary MEP throughout pregnancy were positively associated with weight gain. Accordingly, the risk of impaired glucose tolerance (IGT) increased with high levels of this phthalate metabolite at the second trimester [91,92]. 

More recently, Shaffer et al. found that high mean levels (average concentration between first and third trimesters) of maternal urinary MEP significantly increased the odds of IGT and GDM [93]. Similarly, BPA urinary levels at the second trimester of pregnancy were positively associated with post-load glycaemia levels [94]. However, other cohort studies did not confirm the association between EDCs exposure and IGT, showing contrasting results for both BPA [95,96,97,98,99] and phthalates [97]. A possible reason for these controversial outcomes lies in the confounding effect of adiposity. Indeed, in a further analysis of the Lifecodes cohort study, Bellavia et al. investigated the association between first and second trimester BPA urinary levels and post-load glycaemia both in the overall sample and stratifying by BMI categories. Remarkably, although not any significant relationship emerged when considering the full sample, in the overweight/obese subgroup, higher concentrations of BPA at both trimesters were significantly associated with high post-load glycaemia [100]. 

Besides hyperglycaemia, in a prospective study, phthalate exposure in a low-risk cohort of pregnant women has been linked to increased diastolic blood pressure within 20 weeks of gestation and to the development of gestational hypertension and pre-eclampsia in late pregnancy [101].

Long-term adverse outcomes have been observed in mothers with BPA-associated insulin resistance in pregnancy. Specifically, pregnant mice treated with BPA had significantly higher body weights four months after delivery than control mice receiving vehicle [102]. Furthermore, the same authors reported that pregnant mice exposed to BPA developed insulin resistance and glucose intolerance later, along with significant weight gain, compared to controls [103]. Phthalates have been linked to long-term weight gain in the mother as well [104], potentially contributing to the development of metabolic diseases, such as T2D and metabolic syndrome, the well-known long-term complications of GDM [12,105]. There is evidence in the mouse model that BPA exposure in pregnancy targets insulin signalling pathways in peripheral tissues (liver and adipose tissue) by inhibiting the phosphorylation of Akt, therefore inducing impaired glucose homeostasis [102]. Moreover, BPA has a 17-β estradiol-like (E2) effect, as it is able to bind both ERα and ERβ, activating several signalling pathways. Notably, estrogen receptors are widespread throughout different tissues and are known to play a role in the regulation of glucose homeostasis [106]. In the mouse model, chronic administration of both E2 and BPA increased insulin synthesis and release by the pancreatic β-cell, inducing chronic hyperinsulinemia. Chronic hyperinsulinemia eventually led to insulin resistance in this model [107,108]. There is also evidence indicating that BPA increases the activity of PPARγ in adipocytes [109,110].

EDCs target the pancreatic β-cell as well [111]. Given that GDM occurs when the compensative effect of the pancreatic β-cell fails, the interference of EDCs might contribute to the development of hyperglycaemia. Furthermore, inflammation and oxidative stress are thought to be involved in the pathogenesis of insulin resistance in pregnancy. A positive correlation between BPA, inflammation and oxidative stress markers (IL-6, 8-isoprostane and 8-hydroxydeoxyguanosine) was also observed in pregnant women in early pregnancy [112].

### 4.2. Impact on Offspring: Short-Term and Long-Term Outcomes 

Low birth weight is a well-known risk factor for obesity and T2D [113]. Interestingly, EDCs are reported to affect foetal growth and the length of pregnancy in cohort studies. In a study involving 482 pregnancies, levels of maternal urinary DEHP metabolites were inversely associated with foetal growth parameters (femur length, head circumference and weight) [114]. Other authors found that phthalate exposure at the third trimester of gestation was positively linked to the risk of preterm birth as well [115]. Several studies have investigated the relationship between BPA exposure and pregnancy outcomes, with controversial results. Indeed, in the prospective Upstate KIDS study, BPA levels in the blood of 6171 infants after delivery were negatively associated with the length of pregnancy, birth weight and head circumference [116]. Veiga-Lopez et al. observed similar results only regarding birth weight, although gender differences emerged. Indeed, the negative correlation between maternal urinary BPA at the first trimester and birth weight was stronger in pregnancies with a female foetus. Conversely, a significant increase in the lengths of the pregnancies was observed, although more marked when the foetus was a female [117]. In another cohort study, increased BPA concentrations in maternal and cord blood were predictive of a higher risk of low birth weights in male foetuses [118]. However, in a recent meta-analysis of eight studies, no significant correlation emerged between BPA exposure and birth weight [119]. 

Overall, in a large meta-analysis of 13 European cohort studies enrolling 133,957 pregnancies, exposure to EDCs was associated with a significant increase in the risk of low birth weight [120]. 

The molecular mechanisms underlying the possible association between exposure to EDCs during pregnancy and reduced foetal growth are not completely uncovered. The disrupting effect of phthalates is suspected to target placental TR. In mice exposed to the phthalate metabolite DEHP, placental levels of TR mRNA were found to be reduced in small for gestational age progeny [121]. Similarly, the expression of several factors involved in the regulation of placental angiogenesis, such as vascular endothelial growth factor, placental growth factor, insulin-like growth factor-1 and insulin-like growth factor-2, was reduced. In parallel, a lower number of micro-vessels emerged histologically in placentas [121]. Other mechanisms, such as the alteration of patterns of placental micro-RNA expression, DNA methylation and gene imprinting in the placenta, might be involved [82]. Interestingly, it has been observed that phthalates interfere with the expression of multiple genes, including epidermal growth factor, in the placenta at the first trimester by altering DNA methylation [78].

Besides short-term pregnancy outcomes, several cohort studies have focused on the effects of prenatal and early life exposure to EDCs on childhood adiposity. As regards BPA, the main findings are controversial. The Maternal-Infant Research on Environmental Chemicals (MIREC) study enrolled 719 mother-child pairs, and urinary levels of BPA were determined in mothers at 12 weeks of gestation. Anthropometric parameters were obtained at 3.5 years in children, and a positive association between BPA concentration and waist-to-hip ratio was observed [122]. In the RHEA cohort study, relevant sex differences emerged, since maternal BPA in urine was inversely related to BMI in females and positively in males at 4 years of age, whereas increasing postnatal levels of urinary BPA in progeny were significantly predictive of higher BMI, waist circumference and skinfold thickness (an indicator of central adiposity) in both genders [123]. Harley et al. observed that BPA concentration in maternal urine associated inversely with fat mass and BMI only in girls of 9 years of age, whilst urinary BPA measured at 9 years of age correlated positively with BMI, waist circumference and fat mass at the same determination time both in females and in males [124]. The specific time of exposure might therefore explain the different effects of a substance in the same population. However, in other cohort studies, no association between both prenatal and early life levels of BPA and childhood adiposity emerged [122,125]. In the Eden Mother-Child Cohort study, 520 mothers and children (of male sex only) were recruited and followed up for 5 years. Phthalate metabolites during pregnancy were positively linked to BMI at 5 years and to weight increase from 2 to 5 years of age in children [126]. 

The effect of multiple substances has been evaluated as well in a Spanish cohort study of 470 mother-child pairs with a 7-year follow-up, and phthalate exposure during pregnancy was a negative predictor of overweightness at 7 years of age [127]. 

Overall, although the results from cohort studies are not univocal, exposure to EDCs during pregnancy or early in life might have obesogenic properties, possibly playing a role in the development of metabolic diseases many years later. It is recognized that adipocyte dysfunction and inflammation contribute to the development of T2D and GDM [128,129]. Preclinical studies have investigated the action of EDCs on adipocytes. Indeed, adipocyte hypertrophy occurred in the progeny of rats that received BPA during pregnancy, along with a rise in the expression of pro-adipogenic factors. Proliferation of pre-adipocyte induced by BPA has been described in vitro as well [109,110,130]. In cultures of 3T3-L1 pre-adipocytes, the expression of PPARγ was enhanced by BPA, and lipid content in mature cells increased. In parallel, reduced insulin sensitivity and the enhanced expression of both leptin and the pro-inflammatory cytokine IL-6 were observed [110]. In a cohort study, 250 mother-offspring pairs were followed up until 8–14 years. A positive association was found between urinary phthalate metabolites measured in mothers during pregnancy and circulating leptin levels in females and an inverse association with insulin secretion in males at puberty [131]. In the MIREC study, high BPA urinary levels measured at the first trimester of pregnancy were predictive of low adiponectin in cord blood at birth, although only in male foetuses. Furthermore, a significant association between the metabolite of DEHP mono-(3-carboxypropyl)-phthalate and increased circulating leptin levels were observed in males [132]. 

Studies in animal models have reported that BPA exposure during pregnancy leads to the same metabolic alterations induced by high fat diets in offspring later in life, such as hyperglycaemia, IGT and high levels of non-esterified fatty acids [133,134]. This effect might be explained not only by the disruption of adipocyte function but also by the induction of β-cell dysfunction. Indeed, in male offspring of pregnant mice, β-cell inflammation, mitochondrial dysfunction and β-cell death were observed after exposure to BPA, and the damage persisted even in the next generation. Interestingly, gene expression in the β-cell was modified by altering DNA methylation [135]. 

## 5. Conclusions

It is well-established that an unfavourable maternal diet can lead to poor pregnancy outcomes. EDCs are broadly diffused in food, and the risks connected with exposure are suspected to be largely uncovered. Overall, there is evidence that BPA and phthalates are able to affect pregnancy and early life, influencing foetal growth and childhood adiposity. Their disrupting effect is likely to have long-term and transgenerational consequences in the field of metabolic diseases occurrences. Further research is mandatory to clarify the real impact of these substances on the risk of developing GDM and, importantly, to assess whether the limitation of exposure through the adoption of appropriate preventive measures might effectively reduce the incidence of this condition. 

The synergistic effect deriving from exposure to multiple substances at the same time is a key aspect as well. Providing that EDCs are ubiquitous, the interactions between different EDCs and the overall effect should also be considered in real settings, rather than focusing on the actions of a single substance.

Remarkably, several studies have reported sex-specific findings, and more research should also clarify the mechanisms behind gender differences in pregnancy outcomes. 

Finally, long-term follow-up studies are needed to further investigate the association between pregnancy exposure to EDCs and the risk of metabolic dysfunctions in adulthood.
